# SLEEP AND FREQUENT HOSPITALIZATIONS AT THE END OF LIFE FOR INDIVIDUALS WITH DEMENTIA

**DOI:** 10.1093/geroni/igad104.0506

**Published:** 2023-12-21

**Authors:** Karen Klingman, Suzanne Sullivan

**Affiliations:** SUNY Upstate Medical University, Syracuse, New York, United States; SUNY Upstate Medical University, Syracuse, New York, United States

## Abstract

Older adults near end-of-life (EOL) are at risk for experiencing non-beneficial hospitalizations, negatively impacting their quality of life. Reasons for frequent hospitalizations include poorly managed symptoms of progressing chronic illnesses, many of which are exacerbated by poor sleep, and may differ depending on presence of dementia. This study characterized sleep for groups with and without frequent (two-or-more) hospitalizations in the final year of life using the National Health and Aging Trends Study. A subgroup of persons living with dementia (PLWD) was also explored. Means for difficulty falling asleep, trouble falling back to sleep, and use of sleep medications were compared for groups with and without frequent hospitalizations via one-tailed t-tests. Association between dementia and frequent hospitalizations was explored via Pearson Chi-Square. Individuals at EOL (n=529, 56% female, 68% white non-Hispanic, average age 80-85, 57% PLWD) were included, and 22% had frequent hospitalizations. Among the entire sample, those with frequent hospitalizations had worse sleep (more trouble falling back to sleep, p=.032, Cohens-d=.2). Sleep was also worse for those without dementia, but with frequent hospitalizations (difficulty falling back to sleep, Cohens-d=.3, p=.02) compared to those without frequent hospitalizations. There was no association between frequent hospitalization and presence of dementia (chi-square=.868 p=.352). However, the PLWD subgroup had worse sleep than those without dementia (difficulty falling asleep Cohens-d=.15, p=.04; use of sleep medications Cohens-d=.26, p=.002), yet sleep did not differ depending on frequent hospitalizations. These results indicate that further investigation into the relationship between dementia, sleep, and frequent hospitalizations at EOL is warranted.

